# NSPG: An Efficient Posture Generator Based on Null-Space Alteration and Kinetostatics Constraints

**DOI:** 10.3389/frobt.2021.715325

**Published:** 2021-08-10

**Authors:** Luca Rossini, Enrico Mingo Hoffman, Arturo Laurenzi, Nikos G. Tsagarakis

**Affiliations:** ^1^ Humanoids and Human Centred Mechatronics Lab, Istituto Italiano di Tecnologia (IIT), Genova, Italy; ^2^ DIBRIS, Universitá di Genova, Genova, Italy

**Keywords:** whole-body planning, posture generation, humanoid robot, legged robot, hierarchical inverse kinematics, optimization

## Abstract

Most of the locomotion and contact planners for multi-limbed robots rely on a reduction of the search space to improve the performance of their algorithm. Posture generation plays a fundamental role in these types of planners providing a collision-free, statically stable whole-body posture, projected onto the planned contacts. However, posture generation becomes particularly tedious for complex robots moving in cluttered environments, in which feasibility can be hard to accomplish. In this work, we take advantage of the kinematic structure of a multi-limbed robot to present a posture generator based on hierarchical inverse kinematics and contact force optimization, called the null-space posture generator (NSPG), able to efficiently satisfy the aforementioned requisites in short times. A new configuration of the robot is produced through conservatively altering a given nominal posture exploiting the null-space of the contact manifold, satisfying geometrical and kinetostatics constraints. This is achieved through an adaptive random velocity vector generator that lets the robot explore its workspace. To prove the validity and generality of the proposed method, simulations in multiple scenarios are reported employing different robots: a wheeled-legged quadruped and a biped. Specifically, it is shown that the NSPG is particularly suited in complex cluttered scenarios, in which linear collision avoidance and stability constraints may be inefficient due to the high computational cost. In particular, we show an improvement of performances being our method able to generate twice feasible configurations in the same period. A comparison with previous methods has been carried out collecting the obtained results which highlight the benefits of the NSPG. Finally, experiments with the CENTAURO platform, developed at Istituto Italiano di Tecnologia, are carried out showing the applicability of the proposed method to a real corridor scenario.

## 1 Introduction

Achieving autonomous whole-body motion behaviors for humanoid and legged robots still represents a challenging research topic. The capability to take autonomous decisions while walking or manipulating objects is fundamental for complex platforms working in real and uncertain environments, without the need for human supervision and intervention Polverini et al. (2020). Effective motion planning on highly redundant robots with a large number of degrees of freedom (DoFs) requires satisfying multiple objectives and constraints concerning loco-manipulation and stability while avoiding internal (self) and external collisions with the environment. Indeed, the computational cost of a motion planning algorithm dramatically increases depending on the dimension of the state space (i.e., DoF number). For motion planners who directly search on joint space configurations, this could often lead to the impossibility of finding a solution within a reasonable time (a.k.a. *curse of dimensionality*). To overcome this issue, previous works adopt simplifying assumptions to reduce the state space dimension or use a *discrete control space* (i.e., *actions*) Cognetti et al. (2015); Ferrari et al. (2018); Hauser et al. (2008). However, these methods suffer from the trade-off between a small action set, which can reduce the branching factor of the search tree inhibiting specific motions, and a large action set, which increases the branching factor of the search tree that becomes harder to explore. Alternatively, planners based on continuous optimization were used in Deits and Tedrake (2014); Kuindersma et al. (2016); Ratliff et al. (2009), but they do not guarantee *completeness* or *global optimality* and it is non-trivial to generate optimal collision-free trajectories within time frames acceptable for online planning, especially in complex environments.

A further possibility is to use *footstep/multi-contact planners* Bouyarmane and Kheddar (2012); Hauser et al. (2005); Kuffner et al. (2001); Tonneau et al. (2018), which have been widely applied in biped robots. In these approaches, the state space is reduced to consider only the position and orientation of each contact as a working variable. The price to pay is the necessity to move back to the configuration space through a map that associates a whole-body configuration of the robot with a specific set of contacts (i.e., stance) coming from the planner. Indeed, collision with the environment, self-collision, and equilibrium are constraints to be considered when generating a posture projected onto the planned contacts, with the risk of invalidating the sampled state if a feasible configuration cannot be found. Furthermore, the configurations must be generated in such a way that they guarantee a feasible transition motion between them, which is one of the most strict requirements to satisfy. This is done from both stability and collision safeness points of view and will be better explored in the next sections. Hence, footstep and multi-contact planners rely heavily on posture generators, which have to not only satisfy the aforementioned constraints but also be able to generate new postures efficiently.

Addressing these concerns, in this work, we propose a novel posture generator algorithm based on the hierarchical inverse kinematics (HIK), called the *null-space posture generator* (NSPG), able to generate collision-free and statically balanced configurations for arbitrarily complex floating-base robots. In particular, the NSPG exploits the null-space of the robot, which is used to locally correct its posture around a *nominal configuration*, generated starting from the history of previously computed feasible postures. In our method, the previous configuration becomes also the seed configuration of the HIK solver, guaranteeing minimal differences between adjacent postures. If a chain of the robot is in contact with the environment or in self-collision, instead of generating a new whole posture, only the involved kinematic chain is moved to avoid the collision, in the neighborhood of the nominal configuration. Hence, we take advantage of the kinematic structure of multi-limbed robots to restore feasibility. Differently, when the generated posture is statically unstable, only the root link is moved to restore feasibility.

**FIGURE 1:**
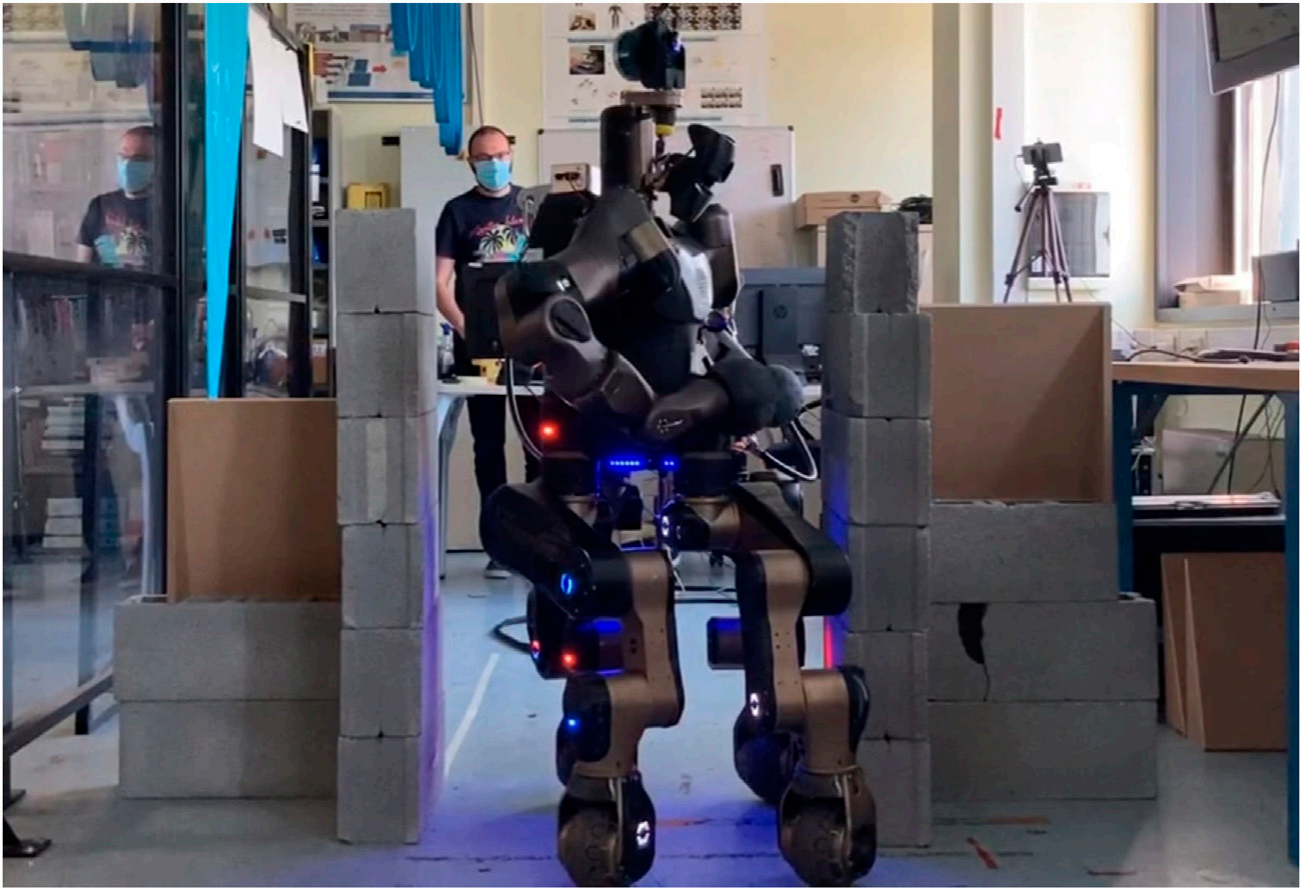
CENTAURO passes through a corridor while the null-space posture generator (NSPG) acts by reshaping its whole-body pose.

The method, compared with previous works, contributes with a smart selection of the seed and nominal configurations for the HIK solver, seeking for a new feasible one inside a small workspace around the nominal configuration whose volume is defined by the parameters of the algorithm. Specifically, it exploits the robot workspace in random directions, moving in the neighborhood of the nominal configuration. This allows the posture generator to look for a feasible configuration instead of discarding the state as soon as the first computed configuration is unfeasible, improving the performance of the planner and the algorithm itself. Last, the number of parameters to be tuned is kept minimum and is not dependent neither on the environment nor on the robot (discussed in detail in Section 5). A similar approach has been used in Yang et al. (2016) in which feasible postures were generated using the IK randomly sampling seed configurations from the balanced manifold. However, stability is checked using the projection of the CoM onto the support polygon drawn by feet, thus not considering non-co-planar contacts which were included in the follow-up work Yang et al. (2017) and Ferrolho et al. (2018). The proposed method is first validated in simulations on the hyper-redundant hybrid wheeled-legged quadrupedal robot CENTAURO Kashiri et al. (2019); Figure 1 and on the biped robot COMAN+ Ruscelli et al. (2020) to prove the generality of the algorithm independently from the number of considered end-effectors or complexity of the robotic platform. Both CENTAURO and COMAN+ are developed at Istituto Italiano di Tecnologia. An experimental assessment of the proposed method is also carried out employing CENTAURO’s perception system (Lidar sensor) for perceiving the environment in front of the robot.

This paper introduces the literature on previous related approaches and novel contributions to posture generation applied to contacts and whole-body motion planning in Section 2. The methodology is explained in Sections 3 and 4 that will be used later for the algorithm description in Section 5. To conclude, Sections 6 and 7 describe the obtained simulation and experimental results and how the performance of the introduced method compares to that of the previous work.

## 2 Related Work

The generation of feasible whole-body configurations for a legged robot coupled with footsteps or multi-contact planners has been widely investigated in the past years. The previous work is based on pre-computed paired forward–inverse dynamic reachability maps (DRM/iDRM) to sample in the reachable workspace those configurations that could accomplish a loco-manipulation task while guaranteeing stability and collision safeness on flat Yang et al. (2017) and inclined Ferrolho et al. (2018) terrains. This method is characterized by big computational and memory costs which are reduced, solving for the upper and lower bodies separately. Additionally, it requires the computation and discretization of the reachable workspace which becomes computationally heavier when the number of contacts increases.

In Hauser et al. (2005), an iterative constraint enforcement (ICE) algorithm was used to generate statically stable and collision-free configurations using the Newton–Raphson method. The generated postures are subject to Cartesian constraints for the contacts and CoM position to guarantee stability, starting from randomly sampled initial configurations. However, random seed configurations do not take into account the problem of minimal displacement between adjacent postures, and this could lead to unfeasibilities during the transition motion.

Other approaches explore fixed-size Gutmann et al. (2005); Deits and Tedrake (2014) or variable-size Buchanan et al. (2019) bounding boxes to find the best collision-free walking posture. This whole-body posture is then projected onto the contacts found during the planning phase. However, these methods do not take advantage of the capability of reshaping the whole body of the robot to facilitate and eventually permit transiting in scenarios where the dimensions of the free passage are closely the physical dimensions of the robot body.

In Bouyarmane and Kheddar (2012), a non-linear optimization has been used to compute IK with static stability, collision avoidance, torque limits, and joint limits as constraints. In this work, no further modifications are carried out when the solver is not able to find a solution, leading to a possible avoidable discard of the sampled contact state.

A different approach was used in Tonneau et al. (2018). The contact planner problem is addressed first by finding a guide path for the floating base in the SE(3) configuration space while satisfying a reachable condition to guarantee collision safeness and workspace reachability of the end-effectors. Then, a sequence of discrete configurations is computed using an iterative algorithm that satisfies a specific contact transition, stability, and collision safeness starting from the root guide path. Ultimately, the contact sequence is retrieved from the configuration sequence. However, this method relies on a pipeline that may suffer from a necessary fine-tuning of its parameters, especially for the effectiveness of the reachable condition which strictly depends on the kinematic characteristics of the robot.

Recently, in Shigematsu et al. (2019), a posture generator has been developed to plan whole-body trajectories for a humanoid robot moving heavy suitcases. The approach is based on a non-linear program where several key postures are optimized all together with the centroidal statics, joint limits, and self-collision constraints. Despite the impressive results obtained on the real platform, the method does not account for environmental collisions, and it still needs several minutes to compute a sequence of configurations.

## 3 Methodology

The NSPG aims to generate collision-free whole-body configurations realizing both kinematic and statics constraints that will be detailed in Sections 3.2 and 3.3. These constraints arise from the contacts that the robot is required to establish with the environment to execute an assigned task and are generally the output of a contact planner (Section 3.1). To this end, we introduce in this section the basic notions that will be used in the following.

### 3.1 Stance Generation

The contact planner defines the pose of the active contacts for the legged robot moving from a start to a goal stance. The basic elements used are as follows:

• A *configuration* $q\in \mathrm{SE}\left(3\right)\times {\mathbb{R}}^{n}=Q$ is an element of the robot configuration space containing the *n* joint positions and the pose of the floating base w.r.t. the inertial frame. We denote as $q_{j}\in {\mathbb{R}}^{n}$ the joint positions. Additionally, Q is partitioned by two sub-sets $Q_{\text{feas}}$ and $Q_{\text{unfeas}}$ containing the feasible and unfeasible configurations, respectively, so that $Q_{\mathrm{feas}}\cap Q_{\text{unfeas}}=\emptyset$.
• A *stance* $\sigma =\left\{c_{1},\ldots \;,c_{k}\right\}$ is a set of *k* contacts where each ck=〈Tc,kw,IDc,k,CTc,k〉 contains the pose of the *k*th contact Tckw w.r.t. the inertial frame, the contact’s name ${\text{ID}}_{\text{c},\text{k}}$, and its type ${\text{CT}}_{\text{c},\text{k}}$ (i.e., point or surface contact).
• A configuration q is *compliant* with a stance *σ* if it realizes all the contact poses specified by *σ*, i.e., k(ci,q)=Tciw for all $c_{i}\in \sigma$, with k being a forward kinematics map that computes the pose of contact $c_{i}$ when the robot is in the configuration q. A pair consisting of a stance *σ* and a compliant configuration q defines a *state s*:

s=〈σ,q〉. (1)

For each state $s_{i}$, given the stance $\sigma_{i}$, the posture generator aims to find a feasible configuration $q_{i}$ compliant with $\sigma_{i}$. The path of stances is in turn found by a generic footstep or multi-contact planner. However, the description of the planner algorithm is out of the scope of this work.

The configuration space velocities associated with the configurations q are denoted $\nu \in {\mathbb{R}}^{n+6}$ that contain the joint space velocities and the linear and angular base velocities:

$$\nu =\left[\begin{array}{c} {\dot{p}}_{b}\\ \omega_{b}\\ {\dot{q}}_{j} \end{array}\right].$$ (2)

In addition, with proper validity functions, we assume to sample stances with poses of the contacts in the workspace of the robots and not inside any obstacle.

### 3.2 Hierarchical IK

The Cartesian velocity vew∈ℝ6 of an end-effector frame ${ℱ}_{e}$ w.r.t. a reference ${ℱ}_{w}$ is related to ν through the relation

vew=Jw,ewν, (3)

where Jw,ew∈ℝ6×(6+n) is the Jacobian^1^ of the frame ${ℱ}_{e}$ w.r.t. ${ℱ}_{w}$ expressed in ${ℱ}_{w}$.

The inverse problem of Eq. 3, a.k.a. *differential inverse kinematics*, permits to compute the configuration velocities $\nu^{\text{*}}$ which realize a desired Cartesian velocity $v_{d}$ for a certain end-effector. The computation of $\nu^{\text{*}}$ is classically found solving a *least-square* problem in the form

$$\nu^{\text{*}}\in \text{argmin}\;{\left\|J\nu -v_{d}\right\|}_{W}^{2},$$ (4)

with $W\in {\mathbb{R}}^{6\times 6}$ being a weight matrix. In order to track Cartesian poses as well, a closed loop IK (CLIK) scheme is often employed where the desired Cartesian velocity $v_{d}$ is set as

$$v_{d}=v_{r}+\lambda e\left(T_{r},T\right),$$ (5)

with $v_{r}$ being a feed-forward Cartesian velocity reference, $T_{r}$ being a reference Cartesian pose, T being the actual Cartesian pose, and *λ* being a gain which ensures exponential convergence of the Cartesian error e(⋅) to zero. The configuration velocities computed using Eq. 4 can be integrated to obtain the new robot configuration, through the integration function I:

$$q_{k}=I\left(q_{k-1},\nu ,dt\right).$$ (6)

The problem in Eq. 4 can be formulated as a quadratic programming (QP) problem with the main advantage of considering equality and inequality *constraints* as well Kanoun et al. (2011):

$$\begin{array}{c} \underset{\nu }{\text{min}}\;{\left|\left|J\nu -v_{d}\right|\right|}_{W}^{2}+\epsilon {\left|\left|\nu \right|\right|}^{2}\\ s.t.\;\;A_{eq}\nu =b_{eq}\text{,}\\ \;\;\;\;\;A\nu \leq b. \end{array}$$ (7)

Furthermore, *hard* priorities between tasks can be enforced in the QP-based IK by means of a cascade of QPs Kanoun et al. (2009) or using particular hierarchical orthogonal decomposition of the aggregated task matrices Escande et al. (2014).

We define the following tasks and constraints:

• **Contact Task** $T_{c}$ that projects the robot into the manifold defined by the contact stances *σ.* For example, the surface contact task is defined as

Tcs:=‖Jcswν−λce(Tc,dw,Tcw)‖2 (8)

• with Jcsw∈ℝ6×(n+6), while the point contact task is defined as

Tcp:=‖Jcpwν−λc(pc,dw−pcw)‖2 (9)

• with Jcpw∈ℝ3×(n+6) and pcw∈ℝ3 being the position of the contact.
• **Postural Task** $T_{\nu }$ that tracks a desired configuration velocity $\nu_{d}$ of the robot. The postural task is defined as

$$T_{\nu }:={\left|\left|\nu -\nu_{d}\right|\right|}^{2}.$$ (10)

• As done in the Cartesian case (5), it is possible to define the desired configuration velocity with a term that tracks a reference robot configuration $q_{r}$:

$$\nu_{d}=\nu_{r}+\lambda_{\nu }e\left(q_{r},q\right).$$ (11)

• **Joint Limits Constraint** $C_{q_{j}}$ permits to take into account hardware joint limits present in the considered robotic platform. The joint limits constraint is an inequality constraint in the form

$$C_{q_{j}}:=\frac{{\underset{-}{q}}_{j}-q_{j}}{\mathrm{dt}}\leq {\overset{\cdot }{q}}_{j}\leq \frac{{\overset{-}{q}}_{j}-q_{j}}{\mathrm{dt}},$$ (12)

• with ${\underset{-}{q}}_{j}\in {\mathbb{R}}^{n}$ and ${\overset{-}{q}}_{j}\in {\mathbb{R}}^{n}$, respectively, being the lower and upper joint limits. dt is the integration time used in Eq. 6.

We organize these tasks and constraints in the following stack S:

$$S:=\left[\left(\overset{k}{\underset{i=1}{\sum }}T_{c,i}\right)/T_{\nu }\right]\ll C_{q_{j}},$$ (13)

where the “∑” symbol means that all the contact tasks are summed at the same priority level and the “/” symbol means that the postural task acts in the null-space of the contact tasks and is, hence, the “hierarchical” term in HIK. The “≪” symbol means that all the tasks are subject to the joint limits constraint. This formulation, known as *math of tasks*, follows the work done in Mingo Hoffman and Tsagarakis (2021).

### 3.3 Centroidal Statics

To grant quasi-static stability for a given robot configuration $q_{i}$, compliant with a stance $\sigma_{i}$, a critical role is played by the interaction forces. Static stability is checked solving another QP based on the stances’ information of contact position $p_{c}$, its associated normal $n_{c}$, and CoM position $p_{\text{CoM}}$ computed from the configuration to be checked.

The resulting QP in the variables $x=F_{c}$, with $F_{c}$ being all the contact wrenches w.r.t. the inertial frame^2^, is formulated as

$$\underset{x}{\text{min}}{\left|\left|mg+G_{\text{CD}}F_{c}\right|\right|}_{W_{1}}^{2}+{\left\|F_{c}\right\|}_{W_{2}}^{2}$$ (14a)

s.t.

$$\underset{-}{F}\leq F_{c}\leq \overset{-}{F},$$ (14b)

$$CF_{c}\leq 0;$$ (14c)

$\text{if}\;c_{i}\;\text{is}\;\text{the}\;\text{surface}\;\text{contact,}$

$$PF_{c,i}\leq 0,$$ (14d)

$$NF_{c,i}\leq 0.$$ (14e)

The first term in Eq. 14a ensures static stability, under quasi-static conditions, based on the centroidal statics (CS) of the robot, where the terms $g\in {\mathbb{R}}^{6}$ and m∈ℝ are the vector of the gravity acceleration and momentum variation and the mass of the robot, respectively, and $G_{\text{CD}}\in {\mathbb{R}}^{6\times k}$ is the centroidal dynamics grasp matrix:

$$G_{\text{CD}}=\left[\begin{array}{ccc} I_{3} & \cdots & I_{3}\\ S_{\times }\left(p_{\text{CoM}}-p_{c,1}\right) & \cdots & S_{\times }\left(p_{\text{CoM}}-p_{c,k}\right) \end{array}\right],$$ (15)

with $S_{\times }$ being the skew-symmetric matrix operator.

This reduced description assumes fixed contact placements with associated linearized friction models and unilaterality of the contact force Eq. 14c, center of pressure (CoP) inside the contact surface Eq. 14d, and bounded contact yaw torque Eq. 14e Caron et al. (2015) ^3^, to obtain the interaction forces required to compensate for gravity, achieving static balancing and non-slippage of surface contacts. Matrices C, P, and N are expressed as $C=C_{i}\cdot R_{\text{adj}}$, $N=N_{i}\cdot R_{\text{adj}}$, and $P=P_{i}\cdot R_{\text{adj}}$ with

$$C_{i}=\left[\begin{array}{ccccc} 1 & 0 & -\mu_{i} & | & \\ -1 & 0 & -\mu_{i} & | & \\ 0 & 1 & -\mu_{i} & | & 0_{5\times 3}\\ 0 & -1 & -\mu_{i} & | & \\ 0 & 0 & -1 & | & \end{array}\right],$$ (16a)

$$P_{i}=\left[\begin{array}{cccccc} 0 & 0 & x & 0 & 1 & 0\\ 0 & 0 & -x & 0 & -1 & 0\\ 0 & 0 & y & -1 & 0 & 0\\ 0 & 0 & -y & 1 & 0 & 0 \end{array}\right],$$ (16b)

$$N_{i}=\left[\begin{array}{cccccc} -y & -x & -\mu \left(x+y\right) & -\mu & -\mu & 1\\ -y & x & -\mu \left(x+y\right) & -\mu & \mu & 1\\ y & -x & -\mu \left(x+y\right) & \mu & -\mu & 1\\ y & x & -\mu \left(x+y\right) & \mu & \mu & 1\\ -y & -x & -\mu \left(x+y\right) & \mu & \mu & -1\\ -y & x & -\mu \left(x+y\right) & \mu & -\mu & -1\\ y & -x & -\mu \left(x+y\right) & -\mu & \mu & -1\\ y & x & -\mu \left(x+y\right) & -\mu & -\mu & -1 \end{array}\right],$$ (16c)

Radj,i=[Riw03×303×3Riw], (16d)

where $C_{i}$ and $P_{i}$ are the coefficient matrices of the constraint inequalities expressed in the local force frame and $R_{\text{adj},\text{i}}$ is the adjoint rotation matrix that transforms the wrench from the *i*th local frame ${ℱ}_{i}$ to the inertial frame ${ℱ}_{w}$, computed from the contact normal $n_{c,i}$. In particular, $\mu_{i}$ is the static friction coefficient associated with the *i*th contact, *x* and *y* are half the size of the surface contact^4^, and Riw is the rotation matrix that moves from the *i*th contact frame ${ℱ}_{i}$ to the inertial frame ${ℱ}_{w}$.

**FIGURE 2:**
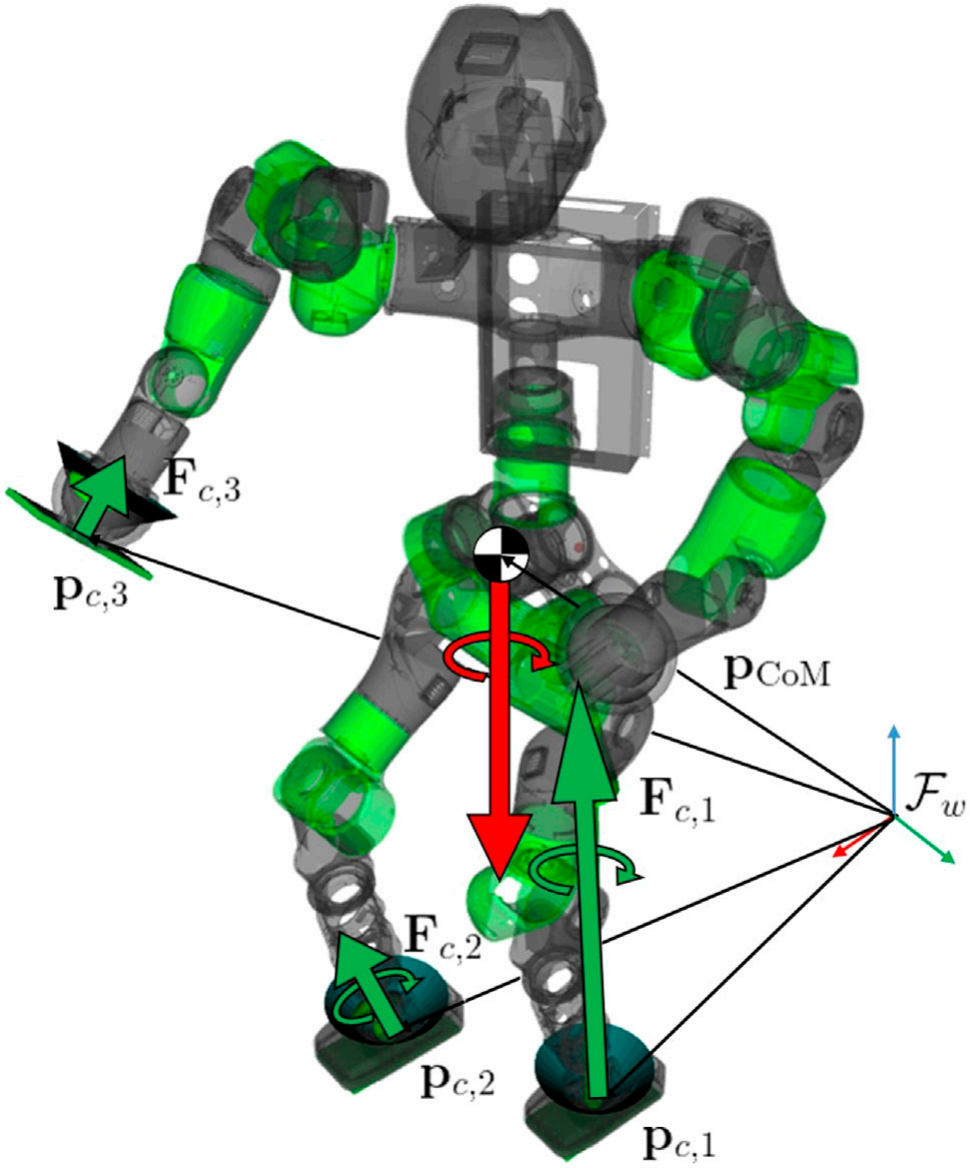
Centroidal statics and robot configuration starting from planned contacts. The green arrows represent the contact forces and torques. These lasts are limited to the surface contacts only (feet). The friction cones are also shown for the three active contacts. The red arrows represent the weight force and the derivative of the angular momentum exerted directly on the CoM of the robot. Position vectors of each contact and CoM are highlighted w.r.t. the inertial frame ${ℱ}_{w}$.

It is worth noticing that modeling a surface contact using forces and moments, together with the constraints Eqs. 14c–e, permits to save variables and constraints. Assuming four contact points per surface contact leads to a total of 12 pure contact force variables and 20 constraints in the form of Eq. 16a. On the contrary, assuming a single wrench leads to 6 variables to describe contact forces and torques and 17 constraints. A graphical representation of the centroidal statics’ components is given in Figure 2.

The residual of the first term in the cost function Eq. 14a is used to decide whether a configuration is stable or not when satisfying a specific stance, depending on a threshold value.

## 4 Generating Transition Configurations

**FIGURE 3:**
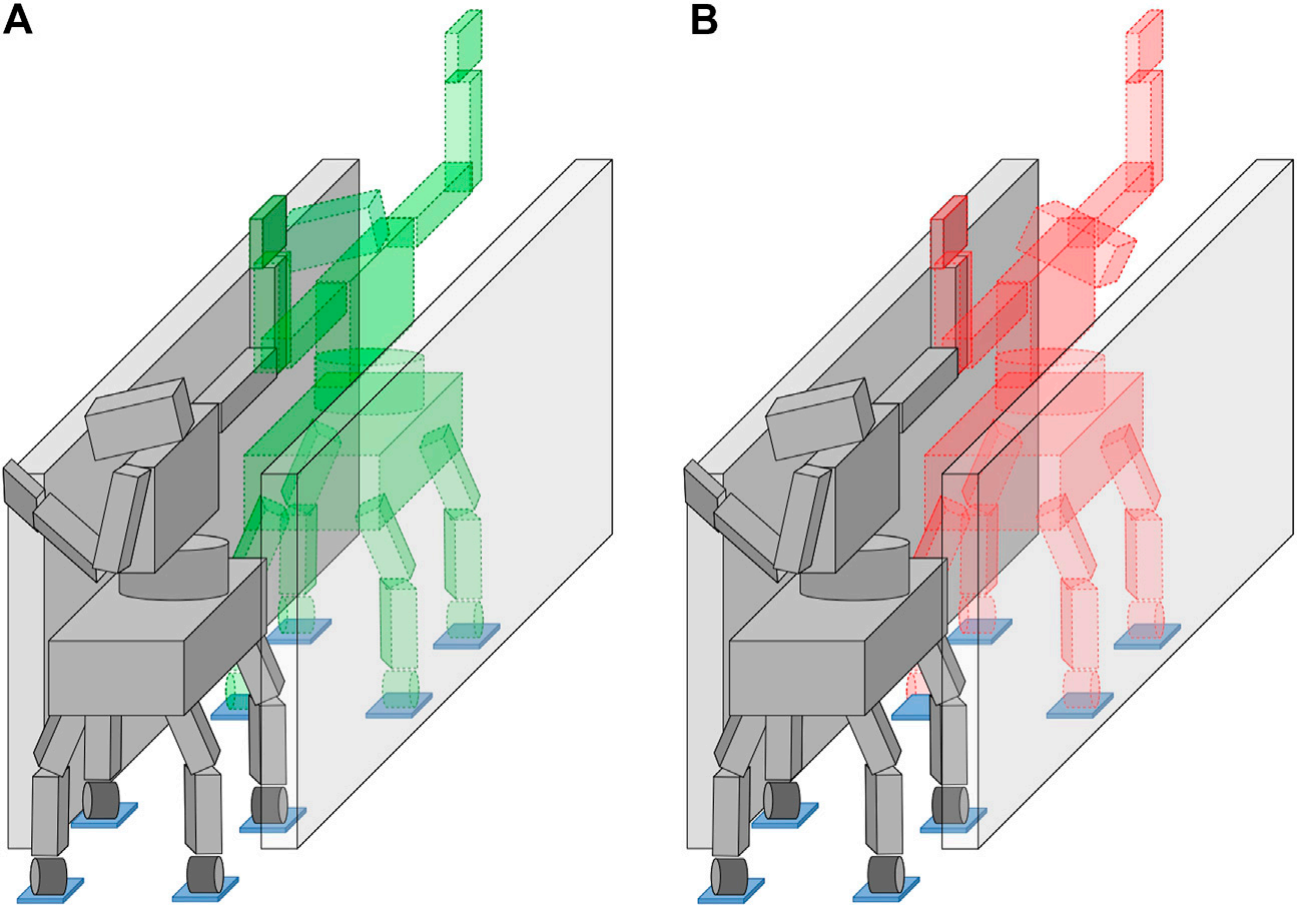
An example of how generating adjacent poses with large differences can lead to an unfeasible colliding transition during their connection. Starting from the same robot configuration in gray, the next posture is generated minimizing the differences w.r.t. the parent state, on the left, or facing the opposite side of the corridor with the upper body, on the right. Even though all the generated poses are feasible, the right scenario will lead to a collision with the environment while connecting the two consecutive states.

In a contact planner application case, a feasible sequence of adjacent postures is required to be connectable in order to build a configuration path that moves the robot safely from a start configuration $q_{\text{start}}$ to a goal configuration $q_{\text{goal}}$. In particular, two configurations $q_{i}$ and $q_{i+1}$ are connectable if there exists a continuous path φ(l) satisfying $\sigma_{i}$, $\sigma_{i+1}$, and the requirements of stability and collision avoidance. Furthermore, a local planner interpolator guarantees a feasible trajectory between two consecutive configurations. Having defined $Q_{\sigma_{i}}$, the set of all configurations that satisfies $\sigma_{i}$, we can say that consecutive configurations are connectable if $\exists \;\varphi :\left[0,l\right]\to Q_{\text{free}}$ such that

$$\varphi \in C^{0}$$ (17a)

$$\varphi \left(0\right)\in Q_{\sigma_{i}}$$ (17b)

$$\varphi \left(l\right)\in Q_{\sigma_{i+1}},$$ (17c)

with $C^{0}$ being the set of continuous functions. Collision avoidance is sought generating similar adjacent poses, thus minimizing the transition motion that moves the robot from $q_{i}$ to $q_{i+1}$. In order to better understand this last requirement, imagine a robot side-walking in a narrow space. In this scenario, feasible postures can be the one with the robot facing both leftward and rightward. However, if two adjacent $\sigma_{i}$ and $\sigma_{i+1}$ contain configurations that face opposite sides of the narrow passage, the transition motion between $q_{i}$ and $q_{i+1}$ will probably collide with the environment, see Figure 3. We solve this issue using the parent state’s configuration $q_{i-1}$ as a nominal configuration for the generation of $q_{i}$, thus forcing $q_{i}$ to be in a small neighborhood of $q_{i-1}$ (Section 5). This assumption works in the hypothesis of small changes of the environment seen by the robot, which covers the most of the considered scenarios. Indeed, when moving in such an environment, the previous feasible configuration is a first good guess to generate the next feasible configuration. In this way, any transition motion is generated only if required.

Furthermore, in the assumption that near stances differ by exactly one active contact, the generated configuration $q_{i}$ must be statically stable w.r.t. the minimum contact number stance between $\sigma_{i-1}$ and $\sigma_{i}$ to guarantee the existence of a stable trajectory between the two stances Escande et al. (2013).

## 5 Null-Space Posture Generator

**FIGURE 4:**
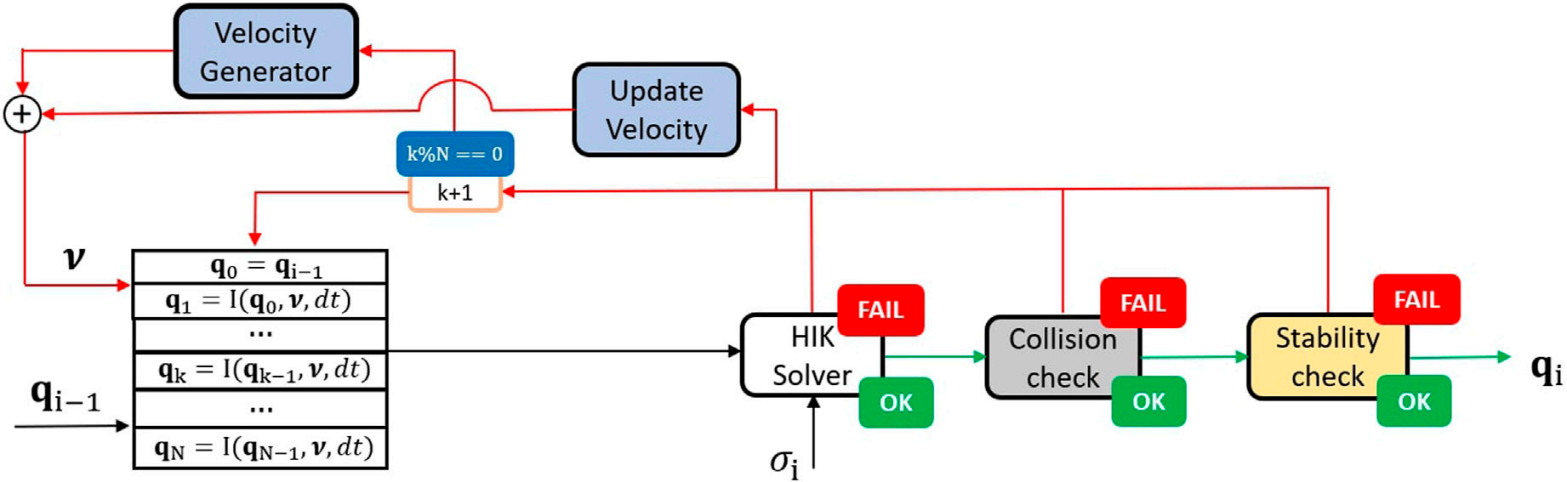
NSPG flow diagram: the configuration $q_{\text{i}-1}$ is used as a nominal posture for the HIK solver, and at each iteration, it is modified depending on the random velocity vector ν generated, until a feasible configuration is found. At each iteration, the velocity vector ν is updated following Section 5.4. Notice that the nominal posture is reset to $q_{\mathrm{i-1}}$ every N iterations. $\sigma_{i}$ defines the manifold for the HIK solver, while green and red arrows define the flow when the validity checks and the HIK projector succeed or fail, respectively.

Our approach is based on a complete reshape of the robot configuration, obtained by adjusting the pose of kinematic chains in a collision, or moving the root link to recover the static stability, in the null-space of the Cartesian (contact) tasks. Specifically, each detected unfeasibility will generate random velocity components aiming to recover feasibility. This section follows a pipeline going through each component of the algorithm: first, the nominal configuration and the random velocity vector ν are generated. The NSPG exploits the neighborhood of the nominal configuration in the random direction defined by ν. Then, the procedure to adapt the velocity vector and the candidate configuration procedures are described. The strategy used is described in Algorithm 1, while a graphical representation is given in Figure 4.

**ALGORITHM 1:** NSPG( ).

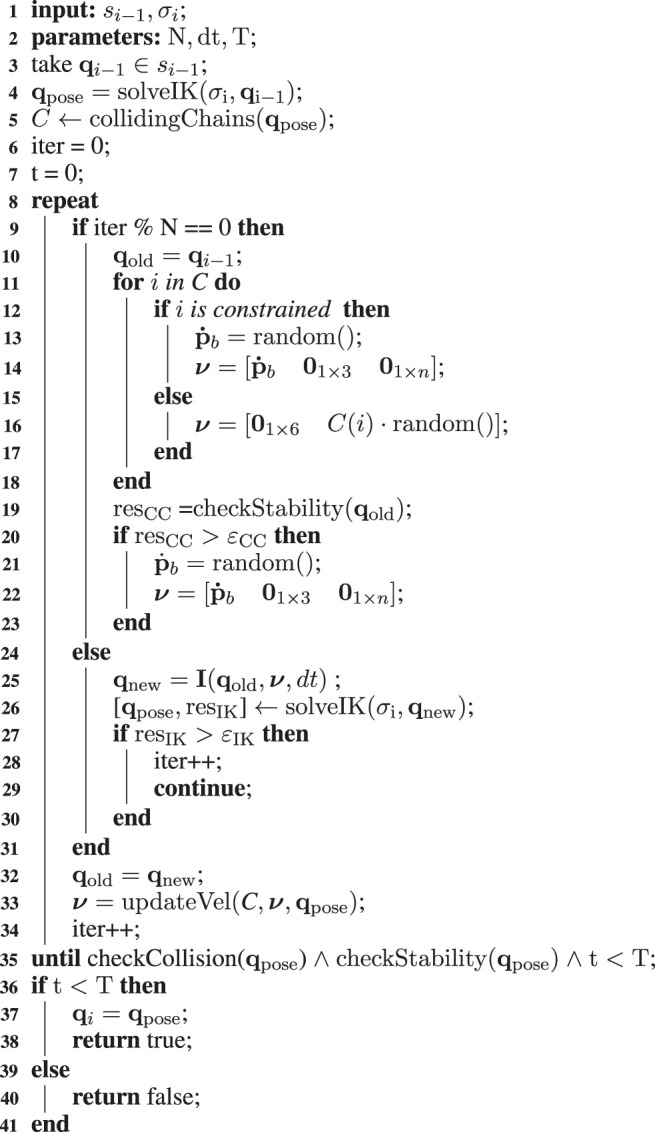

### 5.1 Nominal Configuration Generation

First, the candidate configuration $q_{\text{pose}}$ is computed projecting the *seed configuration* $q_{i-1}$ onto the manifold defined by the stance $\sigma_{i}$. The projection is performed by the HIK solving the stack in Eq. 13 with the same $q_{i-1}$ used as a reference for the postural task (line 4)^5^. In the case the first candidate configuration $q_{\text{pose}}$ is feasible, and no further adjustment is required, the NSPG will return $q_{\text{i}}=q_{\text{pose}}$. Oppositely, $q_{\text{pose}}$ will be used as the nominal configuration in which neighborhood the NSPG will look for a new feasible configuration.

### 5.2 Adaptive Random Velocity Vector Generation

In the following, the procedure to generate the random velocity vector ν will be described, depending on unfeasibility.

#### 5.2.1 Collisions

In this phase, we take advantage of the kinematic structure of a multi-limbed robot to avoid collisions while keeping low differences between adjacent configurations. Indeed, the motion of a kinematic chain is independent concerning the others, assuming that its end-effector is not constrained onto a contact pose. In this way, we can freely move only the chain(s) involved in unfeasibility, preventing avoidable motions that could lead to other collisions.

In detail, when $q_{\text{pose}}$ is in (self-)collision, the colliding kinematic chains are detected (line 5) and collected into the set *C*. The kinematic chain is defined as the set of links and joints moving from the tip link (i.e., end-effector) to the base link. For each of the joints belonging to those chains, a random bounded velocity vector is generated as written in line 16. The random velocity vector comes from a uniform random distribution bounded between the joint velocity limits. C(i) returns a 1×n vector that extracts the aforementioned joints from the whole joints’ list.

It is worth mentioning that collisions/self-collisions can be avoided as well as integrating specific cost functions or constraints in the HIK Fang et al. (2015); Stasse et al. (2008). Despite appealing, this inclusion may have a non-negligible computational cost which is avoidable when HIK is used as a posture generator. The increase in computational cost grows together with the complexity of the surrounding environment which makes the active set computationally expensive. For kinematic chains fully constrained onto the set of contacts defined by $\sigma_{i}$, their reshape is obtained through a linear displacement of the root link as shown in line 14.

With this methodology, the NSPG moves only the joints involved in unfeasibility preventing avoidable motions of the rest of the body.

#### 5.2.2 Stability

A second scenario occurs when $q_{\text{pose}}$ is not statically stable (line 19). The static stability is checked solving the QP problem as written in line 14 comparing the residual of the first term of the cost function with a threshold value $\varepsilon_{\text{CS}}$.

In this case, linear velocities of the root link are generated from a uniform random distribution bounded between two arbitrarily big numbers ($\pm 50\frac{m}{s}$ in our case). This limit value is decided to be arbitrarily big since the postural task is at the lowest level of the HIK solver. Indeed, to obtain a visible motion of the base link, when moving the floating base to recover stability with the specific parameter set chosen, it has to be moved fast enough.

It has been chosen to move the root link instead of the CoM directly since a motion of the latter could imply an undesired whole-body motion involving non-colliding kinematic chains or deviating the motion of the colliding ones unpredictably.

### 5.3 Candidate Configuration Update

The postural task reference is then updated according to the new velocity vector (line 25), and the HIK is solved generating a new robot configuration $q_{\text{pose}}$ according to the new postural reference $q_{\text{new}}$ (line 26). This procedure is repeated every N iterations, until a feasible pose is found, according to $\varepsilon_{\text{IK}}$ and $\varepsilon_{\text{CS}}$ which are chosen to be sufficiently small to guarantee the stable configurations, well-projected onto the contact manifold. The algorithm exploits the robot workspace in the direction defined by ν for N iterations, after which the reference posture of the robot is reset to the starting one $q_{i-1}$ (line 10).

### 5.4 Velocity Vector Adaptation

While running, the algorithm will generate configurations with arbitrarily small differences which depends on its parameters and velocity vector ν.

Collision and stability checks strictly depend on the current candidate configuration of the robot that changes at each iteration of the algorithm. Thus, the velocity vector ν must be updated and adapted depending on the state of the robot throughout each iteration.

Specifically, at each iteration, the colliding chains are updated, and two new sub-sets are defined:

$$C_{\text{new}}\leftarrow \text{collidingChains}\left(q_{\text{pose}}\right),$$ (18a)

$$C_{\text{more}}=C_{\text{new}}\mathrm{\setminus C},$$ (18b)

$$C_{\text{less}}=\mathrm{C\setminus}C_{\text{new}},$$ (18c)

with $C_{\text{more}}$ and $C_{\text{less}}$ containing the set of the new and old colliding chains, respectively, depending on the new candidate configuration. In correspondence with $C_{\text{more}}$, random velocity components are added to ν, while velocity components are removed depending on $C_{\text{less}}$:

$$\nu +=\left[\begin{array}{cc} 0_{1\times 6} & C_{\text{more}}\left(i\right)\cdot \text{random}\left(\right) \end{array}\right],$$ (19a)

$$\nu =\left[\begin{array}{ccc} {\dot{p}}_{b} & 0_{1\times 3} & C_{\text{less}}\left(i\right)\cdot {\dot{q}}_{j} \end{array}\right],$$ (19b)

with $C_{\text{more}}\left(i\right)$ returning a 1×n vector containing 1 in correspondence with the joints belonging to the new colliding chains and 0 elsewhere, while $C_{\text{less}}\left(i\right)$ returns a 1×n vector containing 0 in correspondence with the old colliding chains’ joints and 1 elsewhere. ${\dot{q}}_{j}$ is the old joint velocity vector for the actuated joints, and the “⋅” operator defines a component-wise product between two vectors of the same size. Additionally, stability can be lost or recovered while generating new candidate configurations. In these cases, the velocity vector must be updated adding or removing velocity components corresponding to linear root link velocities as follows:

$${\dot{p}}_{b}=\text{random}\left(\right),$$ (20a)

$$\nu +=\left[\begin{array}{ccc} {\dot{p}}_{b} & 0_{1\times 3} & 0_{1\times n} \end{array}\right],$$ (20b)

$$\nu =\left[\begin{array}{cc} 0_{1\times 6} & {\dot{q}}_{j} \end{array}\right],$$ (20c)

At each iteration, this method looks for a feasible configuration inside a maximum allowable workspace around the nominal configuration. The maximum volume of the robot workspace is defined by the parameters dt and N, multiplied times the maximum velocity vector $\nu_{max}$ containing the absolute value of the velocity limits of each joint. The bigger these values, the bigger the maximum explorable workspace, allowing for bigger differences between adjacent configurations. While the maximum velocity vector strictly depends on the mechanical characteristics of the robot, the two parameters dt and N were tuned after several trials with different parameter sets, picking the best obtained result. The NSPG algorithm moves only the joints involved in the unfeasibility of about a quantity that depends on the NSPG parameters, listed in Table 1. Increasing N allows the robot to explore a larger range of motion around the nominal configuration. The parameter dt is the integration time involved in line 25: the smaller this parameter, the smaller the motion between two generated configurations during a single call of the NSPG. In addition, keeping N constant, the integration time dt will also influence the maximum range of motion around the nominal configuration. Ultimately, the timeout T sets a time threshold for the search of a feasible configuration.

**TABLE 1:** NSPG parameters.

N	Reset condition
Dt	Integration time
T	NSPG timeout
$\varepsilon_{\text{CS}}$	Centroidal statics threshold
$\varepsilon_{\text{IK}}$	IK threshold

## 6 Results

The proposed NSPG algorithm has been tested in two scenarios with increasing difficulty, applied on two different types of legged hyper-redundant robots: the hybrid wheeled-legged quadrupedal robot CENTAURO and the biped robot COMAN+. CENTAURO is a robot with 39 DoFs split between a quadrupedal lower body and a bimanual humanoid upper body, while COMAN+ is a biped humanoid robot with 28 DoFs.

The first considered scenario consists of multiple tiles, placed at different heights and orientations, where the robot has to step on or place its limbs, while the second one is a narrow corridor on a flat terrain. Additionally, an experiment of this last scenario has been carried out on CENTAURO.

Our NSPG implementation is based on the OpenSoT Hoffman et al. (2017) and CartesI/O Laurenzi et al. (2019) frameworks for the computation of the whole-body HIK and centroidal statics QP problems, depicted in Sections 3.2 and 3.3, respectively. In particular, QPs are efficiently solved using well-known QP solvers such as *qpOASES* Ferreau et al. (2014) or *OSQP* Stellato et al. (2020). Collisions are detected exploiting the *Flexible Collision Library* (FCL) Pan et al. (2012) using convex-hull approximations of the links of the robot.

All videos showing the presented simulations and real experiments are included in the material accompanying this paper^6^.

### 6.1 Non-Co-Planar Contact Scenario

The NSPG has been tested in the scenario where an external planner returns a series of feasible stances only. In this case, the contact state can be written as

s=〈σ〉. (21)

**FIGURE 5:**
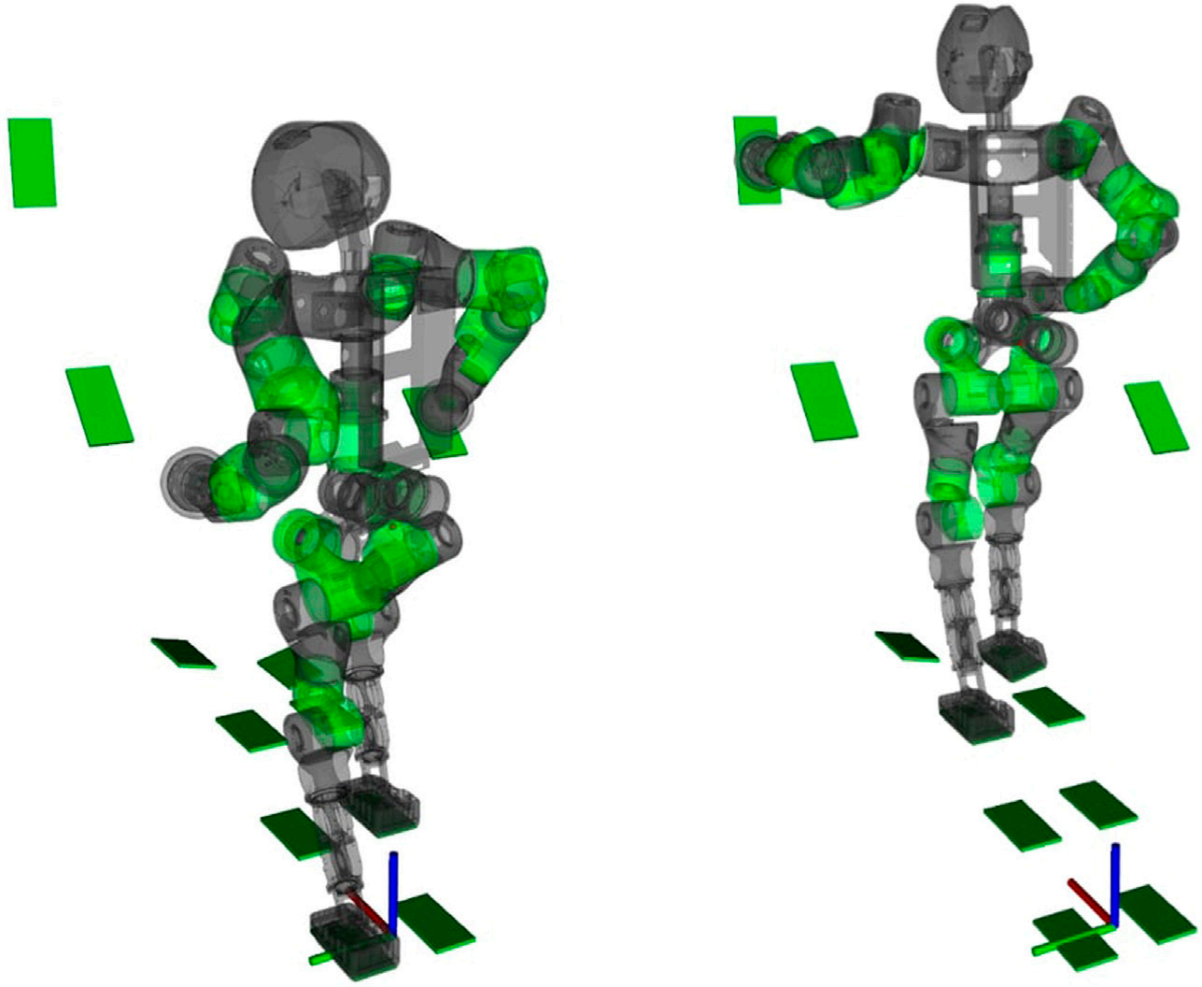
Captures of COMAN+ moving on a sequence of predefined stances while the NSPG generates feasible configurations.

Specifically, the humanoid robot has to climb a stair of three steps on a sequence of 18 stances. The first two steps are flat, while the last two are rotated 0.25 rad along the x-axis (see Figure 5). The stances are such that they respect the principle of connectivity described in Section 4, and the NSPG has to find a series of feasible configurations $q_{i}$, each one corresponding to a specific stance $\sigma_{i}$, in the hypothesis that a feasible configuration exists for each stance. The sequence of stances includes contacts with both hands to enhance static stability. The NSPG is used after the planner and generates a sequence of configurations starting from a sequence of stances. Following the algorithm described in Section 5, the previous configuration $q_{i-1}$ has been used as the nominal configuration for the generation of $q_{i}$, starting from a known *homing* configuration $q_{\text{start}}$.

In this scenario, we want to stress the capability of the NSPG to find collision-free and stable configurations while stepping on non-co-planar stances, using a whole-body approach.

**FIGURE 6:**
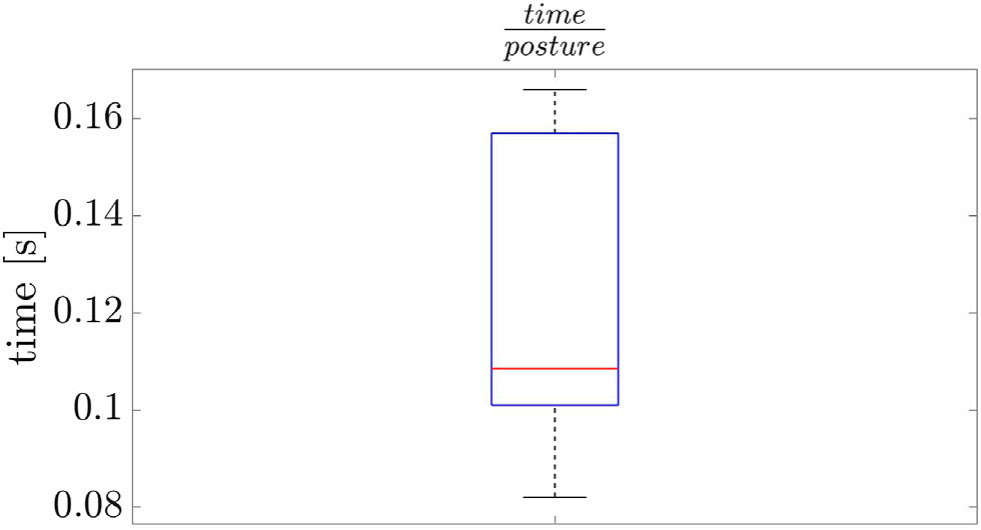
Results from the scenario in Section 6.1.

We analyze the NSPG performance on 10 runs using the same sequence of stances. The results are collected in Figure 6: the NSPG is always able to find all the 18 feasible configurations, changing the active links accordingly, in approximately 2.2 s with an average of 0.12 s for each generated configuration.

### 6.2 Corridor Scenario

The NSPG has been also tested in a particularly tricky scenario for a sample-based planner algorithm: a narrow corridor (Kingston et al. (2018)). In particular, CENTAURO and COMAN+ are asked to traverse a narrow corridor 0.7 m wide and 1.8 m high. Taking advantage of the capability of CENTAURO to roll through the next stance instead of taking a step, its active contacts do not change during the whole planning. In this scenario, CENTAURO proves the effectiveness of our method to generate (self-)collision-free postures being the corridor approximately of the same size of the robot, enhancing the collision occurrances as the robot itself.

When planning with COMAN+ instead, locomotion is achieved by continuously switching between the two feet, i.e., the walking pattern. In particular, a sequence of single and double stances is computed by the planner and connectivity is guaranteed generating single-stance statically stable configurations. Indeed, the next double-stance configuration can be reached if and only if it is single-stance stable, avoiding the robot to go through an unstable region while walking (see Section 4). This is particularly challenging from both stability and collision safeness points of view since the robot has to move its CoM between the two stance feet while moving in the corridor.

In this scenario, the NSPG is used inside a planner routine, implemented using OMPL Şucan et al. (2012), to validate the sampled stances. Every time a new stance $\sigma_{i}$ is sampled, the contact state $s_{i}=\langle \sigma_{i},q_{i}\rangle$ is added to the search tree if the NSPG has been able to compute a feasible whole-body configuration $q_{i}$ in the given time T.

Three parameter setups were tried in this scenario to test how the performance of the NSPG changes. This was evaluated collecting data about the average time employed to find a feasible posture:

$$\bar{t}=\frac{\sum t_{i}}{m},$$ (22)

with $t_{i}$ being the time taken by the NSPG in a single call and *m* being the total NSPG calls. Additionally, the NSPG performance is evaluated considering also its rate of success and the average number of iterations the NSPG takes to find a feasible solution computed as

$$\bar{i}=\frac{\bar{t}}{t_{\text{HIK}}+t_{\text{CS}}+t_{\text{CC}}},$$ (23)

with $t_{\text{HIK}}$, $t_{\text{CS}}$, and $t_{\text{CC}}$ being the average time required by the HIK and the stability/collision check, respectively. The integration time is kept fixed as dt=0.005 seconds, as well as the parameter N=10, to guarantee small differences between adjacent configurations. The timeout T is varied between 0.5, 1, and 2 s. Intuitively, this variation in T should guarantee a higher success rate of the algorithm that is allowed to search a feasible configuration for a longer time. On the contrary, when the mean time to find a single feasible posture increases, the timeout also increases.

An additional statistic has been added considering the causes of failure. Indeed, the NSPG can fail due to collision check and static stability check failure. The necessity to always check both the stability and collision safeness is required by the NSPG to update the velocity vector ν as described in Section 5.4. During the simulations, the number of centroidal statics and collision checks has been collected, and their percentage w.r.t. the total number of fails is shown in Table 2. For CENTAURO, it is observed that the cause of all the failures of the NSPG is (self-)collisions. Furthermore, the quadrupedal structure of CENTAURO guarantees static stability almost in every configuration projected on each stance generated by the planner, and contact states are discarded only when a collision is unavoidable.

**TABLE 2:** Percentages of fail of the centroidal statics check (%CS) and of the collision check (%CC) relative to the total number of fails in the corridor scenario for both CENTAURO and COMAN+.

	%CC	%CS
CENTAURO	100	0.0
COMAN+	43.6	91.6

Differently, when generating configurations with COMAN+, the stability check fails twice the collision check since we are looking for a single-stance stable configuration while moving through a narrow environment. Notice that the sum of the two percentages goes over 100% since the NSPG can fail due to a contemporary fail of the stability and collision checks.

**FIGURE 7:**
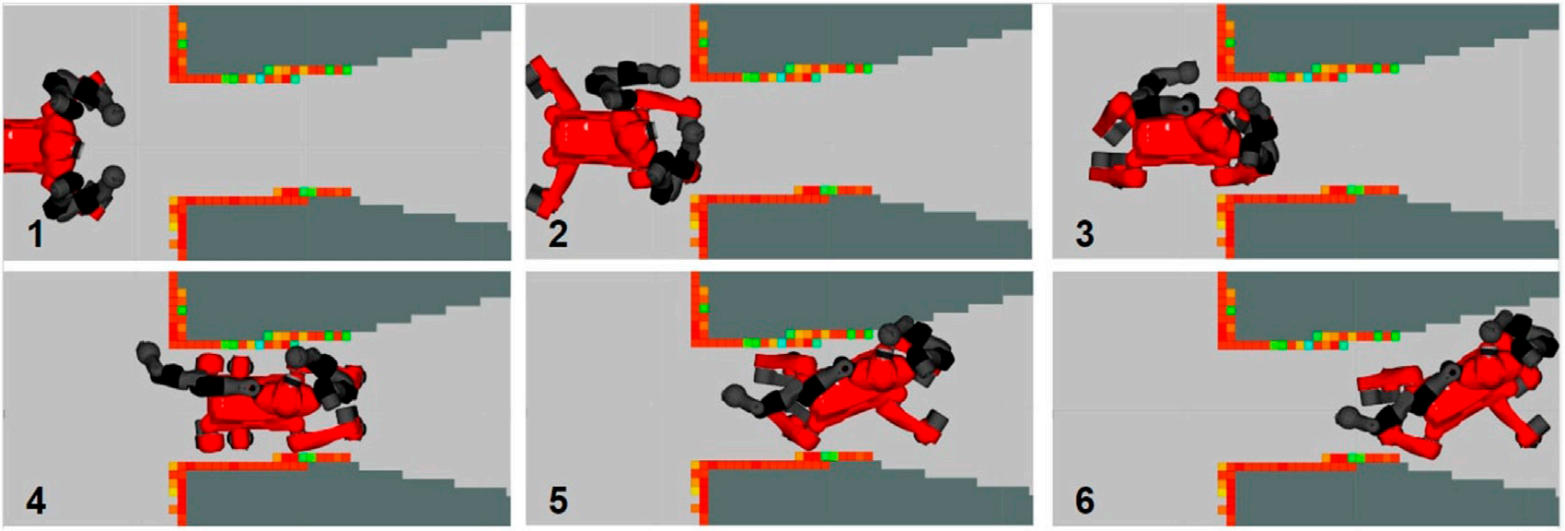
Screenshots of CENTAURO passing through the high narrow corridor.

**FIGURE 8:**
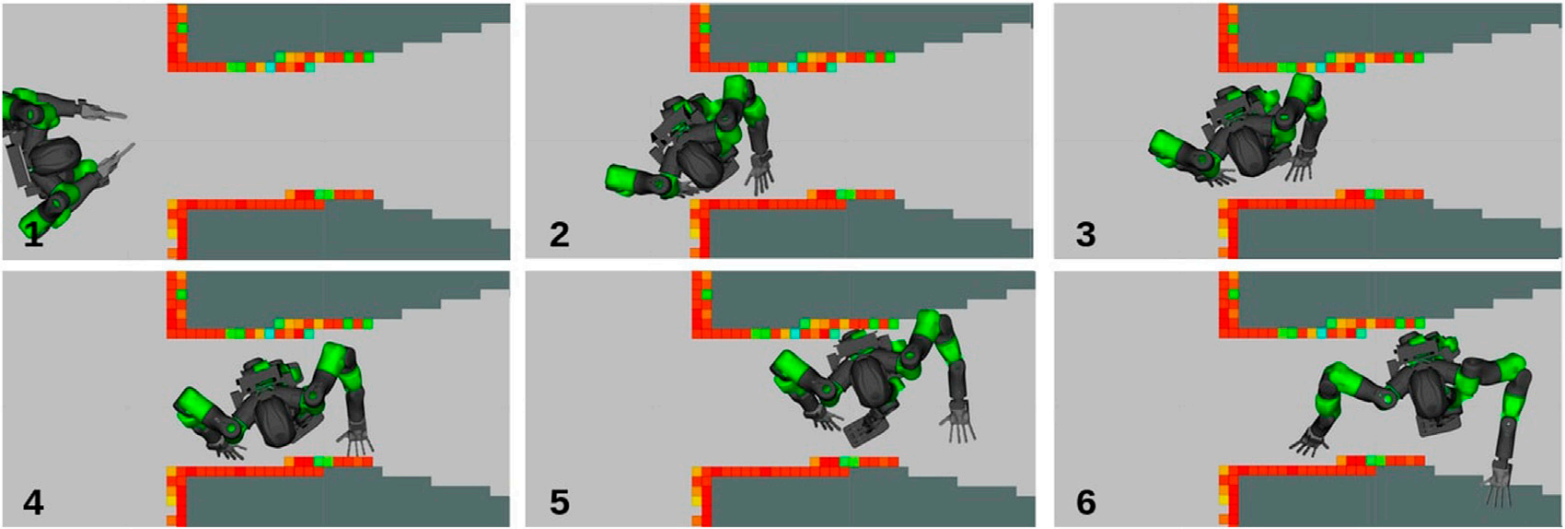
Screenshots of COMAN+ passing through the high narrow corridor.

**FIGURE 9:**
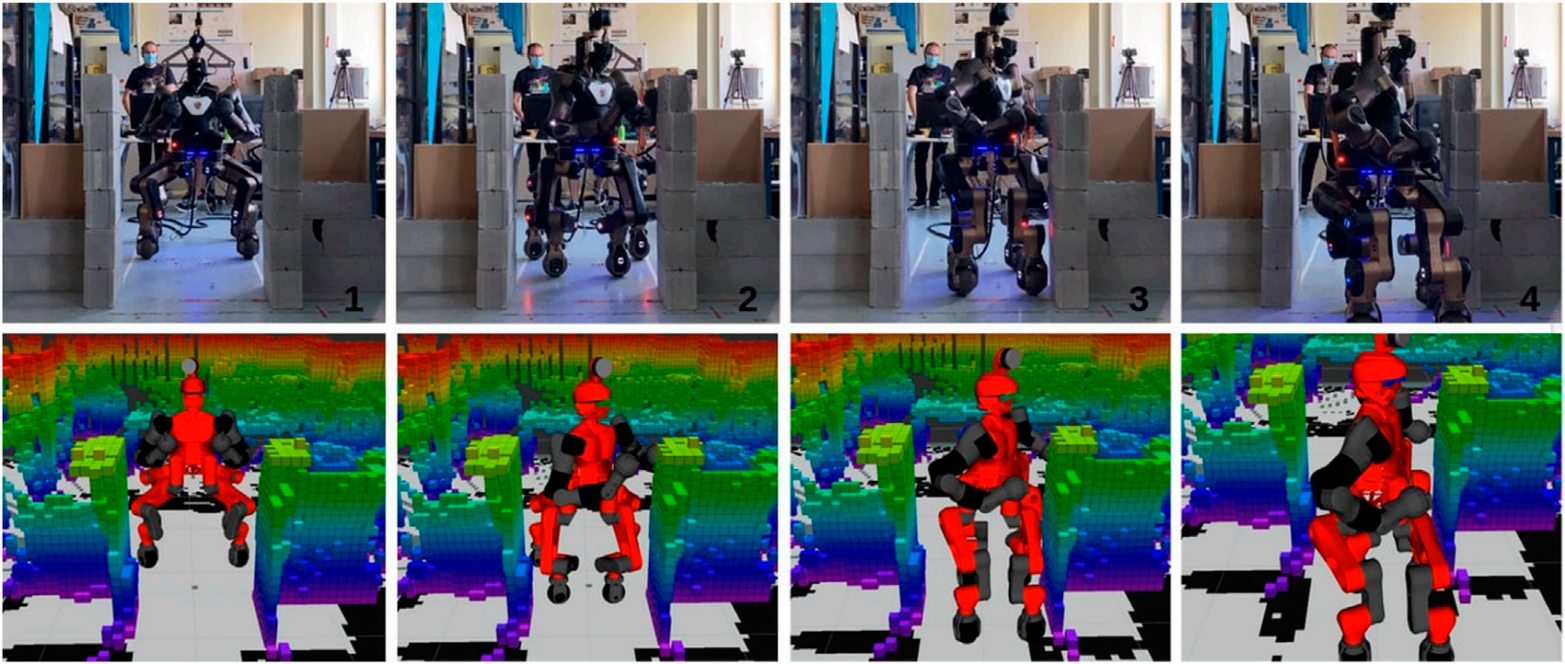
Screenshots from the experiment carried out on a CENTAURO robot. From top left to bottom right, the robot first approaches the narrow corridor keeping the homing position as far as no collisions are detected. As soon as the robot starts entering the corridor, the NSPG reshapes the support polygon and the upper body at the same time.

The results are collected in Table 3, which confirm the observations just done. Screenshots of the simulations with both COMAN+ and CENTAURO are shown in Figure 7 and Figure 8. Real experiments with CENTAURO in this scenario are shown in Figure 9. A complete description of the controller used is given in Appendix 1. In both the simulated and real experiments, the surrounding environment is detected using perception data based on a 3D point cloud generated by a Lidar sensor. The data are collected following the work in Hornung et al. (2013).

**TABLE 3:** Results from the scenario in Section 6.2: dt, N, and T are the three parameters of the NSPG, and ${\bar{t}}_{\text{HIK}}$, ${\bar{t}}_{\text{CS}}$, and ${\bar{t}}_{\text{CC}}$ are the average time required by the HIK solver, the centroidal statics, and the collision check, respectively, averaged on the three experiments, which do not depend on the parameter T. The average number of NSPG iterations to find a feasible solution is shown in the second last column.

	dt	N	T	$\bar{t}$ [s]	${\bar{t}}_{\text{HIK}}$ [ms]	${\bar{t}}_{\text{CS}}$ [ms]	${\bar{t}}_{\text{CC}}$ [ms]	$\bar{i}$	% success
CENTAURO	0.005	10	0.5	0.1248	0.4296	0.3585	0.1301	136	89.9
			1	0.1337				146	95.0
			2	0.3029				330	92.3
COMAN+	0.005	10	0.5	0.1617	0.5173	0.1875	0.2375	172	85.0
			1	0.2161				230	91.6
			1	0.2927				311	93.9

### 6.3 Discussion

The NSPG has been designed to generate whole-body configurations for multi-limbed robots in a particularly complex environment. For instance, (multi-)contact planners generally require a precise representation of an extensive environment, usually obtained through point clouds. As already mentioned, in these scenarios, the use of HIK solvers, coupled with specific constraints for obstacle avoidance, can be computationally heavy since they are based on the computation of the distances between each link of the robot and each obstacle in the environment (i.e., each point of the point cloud). Based on these distances, several methods have been proposed to move the robot away from the singularity. On the contrary, the NSPG does not need any particular strategy to avoid collisions, and the only requirement is the computation of the distances mentioned above. The results in Table 3 show how this computation can be efficiently done even in a complex environment acquired through a Lidar sensor.

To motivate this, an additional simulation with COMAN+ has been run in an environment similar to the one described in Section 6.2. This time, the NSPG does not take into account collisions to generate the random velocity vector ν, but instead an additional constraint for collision avoidance is added to the HIK solver. The constraint is designed following the work done as in, and the results are collected in Table 4. Simulations were run using the same parameter set as in Section 6.2 setting T=1.0s.

**TABLE 4:** Comparison between the results obtained when generating configurations with the NSPG with and without the collision avoidance constraint. In the first case, the random velocity vector is generated and updated depending only on the static stability of the robot. The table contains the same parameters as in Table 3.

Constraint	dt	N	T	$\bar{t}$ [s]	${\bar{t}}_{\text{HIK}}$ [ms]	${\bar{t}}_{\text{CS}}$ [ms]	${\bar{t}}_{\text{CC}}$ [ms]	$\bar{i}$	% success
NO	0.005	10	1.0	0.2161	0.5173	0.1875	0.2375	230	91.6
YES				2.3016	156.8	0.2027	0.0	15	43.7

As expected, when using the linear constraint for collision avoidance in a complex environment detected through a dense point cloud, the time to solve the HIK dramatically increases by three orders of magnitude. The average time for the collision check is dropped to zero, but this improvement is not enough to justify such an increase in computational cost for the HIK and this is reflected in the overall performances of the NSPG. The percentage of success drops to 43.7% since the average time to find a feasible configuration goes over 2 s with a timeout of 1 s. In addition, the average number of iterations for each call of the NSPG decreases to 15 reducing its capability to explore the workspace.

## 7 Conclusion

This work presents a novel algorithm, named the null-space posture generator (NSPG), able to efficiently generate stable and (self-)collision-free whole-body postures for a generic, multi-limbed, floating-base robot, given a sequence of stances. The NSPG has been developed to speed up the whole-body motion planning of complex robotic systems when passing through particularly challenging environments keeping the tuning procedure as light as possible. Indeed, benefits from computation and efficiency points of view have been demonstrated when applying this algorithm in a particularly complex environment compared to previous methods that use the constraint in the HIK to generate statically stable and collision-free configurations. Furthermore, it can also be used independently as a posture generator, given the active contacts as shown in Section 6.1.

Multiple experiments on two profoundly different robotic platforms, COMAN+ and CENTAURO, demonstrated that the NSPG is capable of quickly generating stable and collision-free configurations for a legged robot in contact with the environment, exploiting null-space motions. In particular, CENTAURO represents a challenging platform for planning considering the high number of DoFs. Real experiments were also carried out on CENTAURO using a Lidar sensor to perceive the environment, demonstrating the applicability of the proposed approach to a real scenario.

Our implementation of the NSPG can generate approximately 1,000 configurations per second, guaranteeing a good exploration despite using a light random approach able to adapt while exploiting the robot’s workspace depending on the unfeasibility occurrence.

The proposed method, even if based on a random approach, presents a good level of reliability which is observed in the result obtained in the two considered scenarios. Additionally, it does not require a big effort to tune its parameters, which does not depend on the robotic platform in use, as it has been seen by the general applicability of the algorithm to two profoundly different robotic platforms.

Comparing our results to the recent work proposed in Shigematsu et al. (2019), in the cluttered scenario (Section 6.1), we were able to double the configurations with an average time smaller than three orders of magnitude, guaranteeing minimal differences between adjacent postures.

Future work will involve the use of the NSPG in a multi-contact planner scenario similar to the one used to generate stances in Section 6.1. Furthermore, the stability check could be improved considering centroidal dynamics, allowing higher dynamic motions and enlarging the set of possible feasible configurations and applications, i.e., kinodynamic planning. Additionally, post-processing of the joint trajectory generated should be investigated in order to correct any unfeasibility during the interpolation or to minimize a user-defined cost function (i.e., minimum length path), using the planner output as an initial guess.

## Figures and Tables

**FIGURE 10:**
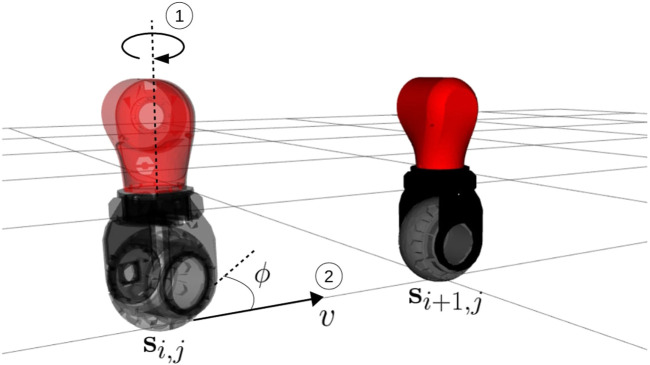
A graphical representation of the wheel control logic. First, the $j^{th}$ wheel $\in s_{i}$ is oriented in the direction of the next state $s_{i+1,j}$ through an ankle rotation, and then the wheel rolls toward the next state position.

## Data Availability

The original contributions presented in the study are included in the article/Supplementary Material, and further inquiries can be directed to the corresponding author.

## Footnotes

1 Here and in what follows, for the sake of brevity of the notation, we do not express the dependence of the matrices on the configuration q.

2 For a point contact, $F_{c,i}\in {\mathbb{R}}^{3}$, while for a surface contact, $F_{c,i}\in {\mathbb{R}}^{6}$.

3 Constraints (14d) and (14e) are considered only for surface contacts.

4 Here, the contact frame is assumed at the center of the surface for simplicity and modeled as rectangular.

5 $q_{\text{start}}\in s_{\text{start}}$ is chosen as the *homing* configuration.

6
https://youtu.be/eEQbz8r5Z3s

## A CENTAURO Controller

In this section, the controller strategy used to replicate the methodology on the real robot CENTAURO will be detailed. Once the discrete collision-free poses have been found, they are connected by using a third-order polynomial interpolation, except for the steering and rolling joints of the wheels, which need special care as detailed hereafter.

Taking advantage of the possibility of rolling toward the next state instead of taking a step, wheels must be first re-oriented in the right direction. For this purpose, a proper controller has been designed as shown in Figure 10: at each state transition, given the initial and final wheel positions from states $\sigma_{i,j}$ and $\sigma_{i+1,j}$, first the wheel is re-oriented toward $s_{i+1,j}$ through a yaw rotation computed as

$$\phi =\text{arctan}\left(\frac{y_{i+1,j}-y_{i,j}}{x_{i+1,j}-x_{i,j}}\right)$$ (24)

where $x_{i,j}$ and $y_{i,j}$ are the coordinates of the $j^{th}$ wheel associated with the $i^{th}$ stance w.r.t. the inertial frame. Subsequently, the wheel is rotated of a quantity equal to

$$\alpha =\frac{d}{r}$$ (25)

where *α* is the rotation that moves the $j^{th}$ wheel from $s_{i,j}$ to $s_{i+1,j}$, *d* is the Euclidean distance between $s_{i,j}$ and $s_{i+1,j}$ computed as $d=||s_{i+1,j}-s_{i,j}|{|}^{2}$, and *r* is the radius of the wheel.

A naive polynomial interpolation cannot guarantee a safe transition of the robot through the discrete configurations. However, in the assumption of small differences between adjacent postures, the probability to encounter unfeasibility drastically decreases. Additionally, an impedance controller guarantees minimum impact consequences in the unluckily event that a small collision occurs while interpolating. Finally, the interpolated trajectory is sent to the robot, and it is tracked through a joint-level impedance controller.
